# Prevalence of multidrug-, extensive drug-, and pandrug-resistant commensal *Escherichia coli* isolated from healthy humans in community settings in low- and middle-income countries: a systematic review and meta-analysis

**DOI:** 10.1080/16549716.2020.1815272

**Published:** 2020-09-10

**Authors:** Nana Adoma Nkansa-Gyamfi, Joseph Kazibwe, Daouda A. K. Traore, Emmanuel Nji

**Affiliations:** a BioStruct-Africa, Vårby, Sweden; b Department of Infectious Disease Epidemiology, Imperial College London, London, UK; c Faculte ′ Des Sciences Et Techniques, Universite ′ Des Sciences, Des Techniques Et Des Technologies De Bamako (USTTB), Bamako, Mali; d Life Sciences Group, Institut Laue- Langevin, Grenoble, France; e School of Life Sciences, Faculty of Natural Sciences, Keele University, Staffordshire, UK

**Keywords:** Antimicrobial Resistance, Antibiotic resistance, multidrug resistance, commensal Escherichia coli, community settings, health policy

## Abstract

**Background:**

The majority of existing studies aimed at investigating the incidence and prevalence of multidrug-resistance by bacteria have been performed in healthcare settings. Relatively few studies have been conducted in community settings, but these have consistently shown a high prevalence of multidrug-resistant bacteria in low- and middle-income countries (LMICs).

**Objectives:**

To provide an appraisal of the evidence on the high prevalence of multidrug-, extensive drug-, and pandrug-resistance in commensal *Escherichia coli* isolates from human sources in community settings in LMICs.

**Methods:**

Using the preferred reporting items for systematic reviews and meta-analyses (PRISMA) guidelines, PubMed, EMBASE, MEDLINE, Web of Science, CINAHL, and Cochrane Library databases were systematically searched with the search string: ‘Enterobacteriaceae’, OR ‘*E. coli*’, OR ‘*Escherichia coli*’, AND ‘antibiotic resistance’, OR ‘antimicrobial resistance’, OR ‘drug-resistance’, AND ‘prevalence’, OR ‘incidence’, OR ‘morbidity’, OR ‘odds ratio’, OR ‘risk ratio’, OR ‘confidence interval’, OR ‘p-value’, OR ‘rate’. Data were extracted and proportional meta-analysis was performed using the Freeman–Tukey transformation random effect model.

**Results:**

The prevalence of multidrug-, extensive drug- and pandrug-resistance were extracted from articles that met our inclusion criteria and pooled together after a systematic screening of 9,369 items. The prevalence of multidrug-resistance was 28% of 14,336 total cases of isolates tested, 95% CI: 23–32. Extensive drug-resistance was 24% of 8,686 total cases of isolates tested, 95% CI: 14–36. Lastly, pandrug-resistance was 5% of 5,670 total cases of isolates tested, 95% CI: 3–8.

**Conclusion:**

This paper provides an appraisal of the evidence on the high prevalence of multidrug-, extensive drug- and pandrug-resistance by commensal *E. coli* in community settings in LMICs. Our results call for greater effort to be placed at the community level in the design of new and improved public health policies to counter the global threat of antibiotic-resistant infections and bacteria.

## Background

Antibiotic resistance (ABR) is one of the most significant global health crises of our time[1–4]. Antibiotics are medicines used to treat bacterial infections [5]. Resistance occurs when the antibiotics become ineffective in blocking one or more of the pathways involved in protein, nucleic acid, cell wall, or folate synthesis, that are essential for bacterial survival. In the USA, more than 2.8 million people are infected by resistant bacteria, and at least 35,000 of these patients die each year [1]. In the European Economic Area, 25,000 and 33,000 people died of infections from resistant bacteria in 2007 and 2015, respectively, indicating that the burden caused by antibiotic resistance is increasing [6,7]. In fact, the World Health Organization predicted that by 2050, the annual number of deaths caused by antimicrobial resistance will increase from 700,000 deaths to 10 million deaths per year globally [8]. The worrying fact is that antibiotic resistance is increasing far more rapidly than the development of new antibiotics [9]. Low- and middle-income countries (LMICs) suffer the most from antibiotic resistance because their healthcare systems lack the resources to contain numerous infectious diseases that are becoming very difficult and expensive to treat due to antibiotic resistance. In high-income countries, stringent policies to limit antibiotic prescribing have been extensively employed to contain the spread of resistant bacteria [10]. Unfortunately, this has not been the case in LMICs, especially at the community level, where antibiotics can be purchase from community pharmacists without a doctor’s prescription. Antibiotic use has been associated with the carriage of resistant commensal *E. coli* in healthy children in community settings worldwide [11].

Extensive antibiotic use leads to an alteration in the gut microbiome [12], and the development of resistance by commensal bacteria to antibiotics through natural selection [13]. One of these commensal bacteria is *Escherichia coli*, one of the main reservoirs for transmitting antibiotic resistance to pathogenic bacteria through the exchange of mobile genetic elements [14,15]. *E. coli* is an Enterobacteriaceae, which is on the World Health Organization (WHO) global critical priority list for research, discovery, and development of new antibiotics. Although LMICs bare the highest-burden of ABR, one cannot exclude the fact that a collective approach involving all sectors is required to reverse its course [1,2,16]. It is well recognized that an emphasis has not been placed at the community level to contain the spread of multidrug-resistant bacteria [17], despite some studies consistently showing the existence of a high incidence and prevalence of multidrug-resistant commensal *E. coli*. If this situation is not addressed, some of the gains made in modern medicine will be reversed due to infections caused by multidrug-resistant bacteria becoming more challenging and expensive to treat. This paper aimed to provide an appraisal on the evidence of a high prevalence of multidrug-, extensive drug-, and pandrug-resistant commensal *E. coli* isolated from healthy humans in LMICs community settings (i.e. locations outside of a hospital, such as schools and homes). These findings should prove useful to researchers, farmers, community pharmacists, policymakers, and advocacy groups among others, to guide the formulation, amendments, and effective implementation of antibiotic resistance-related policies to prevent and contain the spread of multidrug-resistant bacteria.

## Methods

### Design

We systematically identified and synthesized studies that met the inclusion criteria using the preferred reporting items for systematic reviews and meta-analyses (PRISMA) guidelines [18].

### Type of studies

The studies considered for review were cross-sectional, case-control, cohort, and randomized control trials whose primary outcome was the prevalence of multi-drug resistance in commensal *E. coli*.

### Type of participants (study population)

The participants included persons from the general population of all ages and sex, in community settings in LMICs.

### Outcome of interest

Since most of the studies used different definitions for multi-, extensive-, and pandrug resistance, we designed alternative definitions, as described in the primary outcomes sub-section below, to facilitate the inclusion of the studies for meta-analysis.

The primary outcomes were:

• Prevalence of multidrug-resistance, i.e. resistance to one or more agents in two different antibiotic classes (protein, nucleic acid, cell wall, and folate synthesis inhibitors) by commensal *E. coli* in community settings in LMICs.

• Prevalence of extensive drug-resistance, i.e. resistance to one or more agents in three different antibiotic classes by commensal *E. coli* in community settings in LMICs.

• Prevalence of pandrug-resistance, i.e. resistance to one or more agents in four different antibiotic classes by commensal *E. coli* in community settings in LMICs.

The secondary outcomes were odds ratio, risk ratio, rate, 95% confidence interval, and P-value.

### Other inclusion and exclusion criteria

We included all articles published in the English language in LMICs. Articles containing experiments conducted in a country that was an LMICs before transformation into a high-income country, according to the World Bank definition, if any, were also included. Although this was an inclusion criterion, no study was performed in a country that had undergone such transformation.

### Essential data necessary for inclusion

The studies included were those that utilized the Kirby–Bauer disc diffusion or synergy susceptibility test to study and report at least one of the primary outcomes.

### Data sources

The systematic search was performed in PubMed, EMBASE, MEDLINE, Web of Science, CINAHL, and Cochrane Library. Reference lists of selected studies and unpublished data, such as abstracts from conference proceedings, dissertations and theses, were also searched.

### Systematic search strategy

On 10 March 2018, we searched PubMed, EMBASE, MEDLINE, Web of Science, CINAHL, and Cochrane Library using MeSH terms for PubMed and the comparable terms for the other databases. The search was updated on 28 June 2020. The search strings were: ‘E. coli’, OR ‘Escherichia coli’, OR ‘Enterobacteriaceae’, AND ‘antibiotic resistance’, OR ‘antimicrobial resistance’, OR ‘drug resistance’, AND ‘prevalence’, OR ‘incidence’, OR ‘morbidity’, OR ‘odds ratio’, OR ‘risk ratio’, OR ‘confidence interval’, OR ‘P-value’, OR ‘rate’. For PubMed, EMBASE, and MEDLINE, studies performed in humans and written in the English language were selected using the language and species filters. While for Web of Science and the Cochrane Library, additional search words were added to select for species (‘human*’ OR ‘infant*’ OR ‘child*’ OR ‘adolescent*’ OR ‘male*’ OR ‘female’ OR ‘age’ OR “adult*) since there was no sorting filter for species. For CINAHL, neither selection for species nor language was performed. Search words were designed from the different categories in the PICO (Population, Intervention, Comparison and Outcome) format.

### Study selection

The searched articles were exported to the Rayyan software [19], and screening was performed, firstly by title and abstract, and finally by reading the full articles. Two independent researchers screened every article, and a third researcher was available to resolve any disagreements, thereby avoiding selection mistakes.

### Data extraction, statistical analysis and quality assessment

Data were extracted on an Excel spreadsheet. To ensure that no mistakes were made, every article was double-checked by an additional researcher. A third researcher was available to solve any disagreement that arose during the process. Statistical analysis was performed on the Joanna Briggs Institute (JBI) SUMARI [20]. Proportional meta-analysis was performed using a statistical random effect model of Freeman–Tukey. In the analysis, multiple resistance for the different combinations of antibiotics investigated in the studies were included. Quality assessment was performed as previously described [21,22]. Primarily, articles that combined both disc diffusion or synergy tests with polymerase chain reaction (PCR), to confirm both the expression and presence of resistant genes were ranked as ‘high’. Those that utilized only disc diffusion or synergy tests were classified as ‘medium’. Finally, studies with a sample size of less than 15 and those that used single disc diffusion or synergy to determine the expression of resistant genes were classified as ‘low’.

## Results

### Literature search

**Figure 1.**
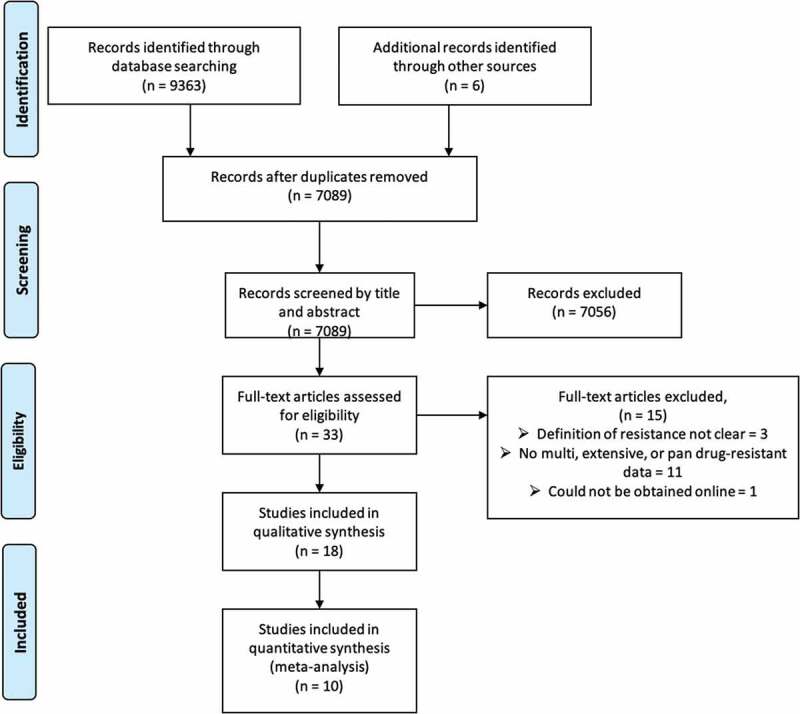
Preferred reporting items for systematic reviews and meta-analyses (PRISMA) flow diagram of the search results and selection of the included studies in the systematic review and meta-analysis. Additional records were identified by screening the reference lists of the included studies

We obtained 9363 articles from the database search and an additional 6 were identified through a review of reference lists of the included articles (Figure 1). A total of 7089 articles were screened by title and abstract after duplicates were removed in order to exclude those that did not meet our inclusion criteria. The full text of 33 articles were assessed for eligibility, and 18 met our inclusion criteria. The remaining 15 articles were excluded because the definition of multiple resistance was not clear (n = 3), did not document any multi-, extensive- or pandrug-resistant data (n = 11), and could not be obtained online (n = 1). A meta-analysis was therefore performed from 10 articles.

### Study location, strength of evidence, and study type

Out of the 10 studies included in the meta-analysis [23–32], six were from Asia and four from South America (Table 1). Since I^2^ statistics for heterogeneity can be misleading [33,34], we performed an alternative assessment, as described previously [21,22]. From the evaluation, five studies were ranked as high quality, while five were ranked as medium. All of the studies were cross-sectional except for two, which were cohort studies.

**Table 1.** Characteristics of studies included for meta-analysis

Study	Age	Number of Isolates	Number of participants	Gender	Type of study	Country	Strength of evidence	Reference
Bartoloni et al. 1998	6–72 months	321	321	Male = 165, female = 158	Cross-sectional	Bolivia	Medium	[23]
Bartoloni et al. 2004	1–77 years	108	108	Male = 61, female = 47	Cross-sectional	Bolivia	High	[24]
Bartoloni et al. 2006	6–72 months	1080	3174	Male = 1627, female = 1546	Cross-sectional	Bolivia	High	[25]
Dyar et al., 2012	6–60 months	738	818	Male = 445, female = 373	Cross-sectional	Vietnam	Medium	[26]
Kalter et al. 2010	3 months–3 years	522	523	-	Cross-sectional	Peru	Medium	[27]
Mamun et al. 1992	1–5 years	649	64	Male = 33, female = 31	Cohort	Bangladesh	High	[28]
Purohit et al. 2017	1–3 years	127	22	-	Cross-sectional	India	High	[29]
Shakya et al. 2013	3–14 years	529	529	Male = 304, female = 225	Cross-sectional	India	Medium	[30]
Singh et al. 2018	1–14 years	550	550	Male = 396, female = 154	Cohort	India	High	[31]
Sahoo et al. 2012	3–9 years	139	417	Male = 230, female = 187	Cross-sectional	India	Medium	[32]

Of the 18 studies included for qualitative synthesis (Figure 1), 10 were excluded for the meta-analysis because the definition of multi-, extensive-, and pandrug-resistance was very vague [23–32] (Table 2). However, there was one study in which parts of the data were included in the meta-analysis while others were not [28]. There were three studies conducted in Africa and five in Asia, with four studies classified as high quality and four medium quality.

**Table 2.** Prevalence and characteristics of included studies not used for meta-analysis

Study	Age	Number of Isolates	Number of participants	Gender	Definition of multi-drug resistance	Pooled prevalence (%)	Lower bound 95% CI	Upper bound 95% CI	Type of study	Country	Strength of evidence	Reference
Aworh et al. 2019	Mean age 30.6 ± 9.7 years	48	122	Male = 121 female = 1	Resistance to three or more classes of antimicrobials	79	66	90	Cross-sectional	Nigeria	Medium	[35]
Al-Dweik and Shehabi, 2009	-	108	108	-	Greater than 2 or 3 of the 14 antimicrobials tested	>2 drugs = 51 >3 drugs = 19	41 12	60 27	Cohort	Jordan	High	[36]
Hoang et al. 2017	20–70 years	103	103	-	Resistance to more than 3 and 5 classes of antimicrobials	>3 classes = 71 >5 classes = 41	62 32	80 51	Cross-sectional	Vietnam	High	[37]
Lamikanra et al. 1996	5–6 years	396	450	-	Resistance to 3, 4, 5, 6, 7, and 8 antibiotics	3 antibiotics = 11 4 antibiotics = 10 5 antibiotics = 10 6 antibiotics = 10 7 antibiotics = 7 8 antibiotics = 8	8 7 7 7 5 5	15 14 14 14 10 10	Cross-sectional	Nigeria	Medium	[38]
Lamikanra et al. 1989	19–25 years	13	35	-	Resistant to at least one other antibiotic	53	22	83	Cross-sectional	Nigeria	Medium	[39]
Mamun et al. 1992	1–5 years	649	64	Male = 33, female = 31	Resistance to 3–5 antibiotics	January to December = 74	60	86	Cohort	Bangladesh	High	[28]
Phongpaichit et al. 2007	12–46 years	143 (non-pig farmers), 7 (pig farmers)	Non-pig farmers (41), Pig farmers (7)	Non-pig farmers male = 211, female = 20	Resistance to at least 1 antimicrobial and multidrug-resistance	Non-pig farmers: at least 1 antimicrobial = 67 multidrug-resistance = 74	60 66	75 81	Cross-sectional	Thailand	High	[40]
Pathak et al. 2012	45 years and above	241	241	Male = 0, female = 241	Resistance to at least 1 or 3 different antibiotics	At least 1 = 94, at least 3 = 35	92 31	96 39	Cross-sectional	India	Medium	[43]

### Age, sample size, gender

For all the 18 included studies (Tables 1 and 2), the age of the participants ranged from 3 months to 77 years, with 7633 participants in total, of which approximately 55% were male and 45% female (Tables 1 and 2).

### High prevalence of multidrug-resistance (MDR) in community settings in low- and middle-income countries

**Figure 2.**
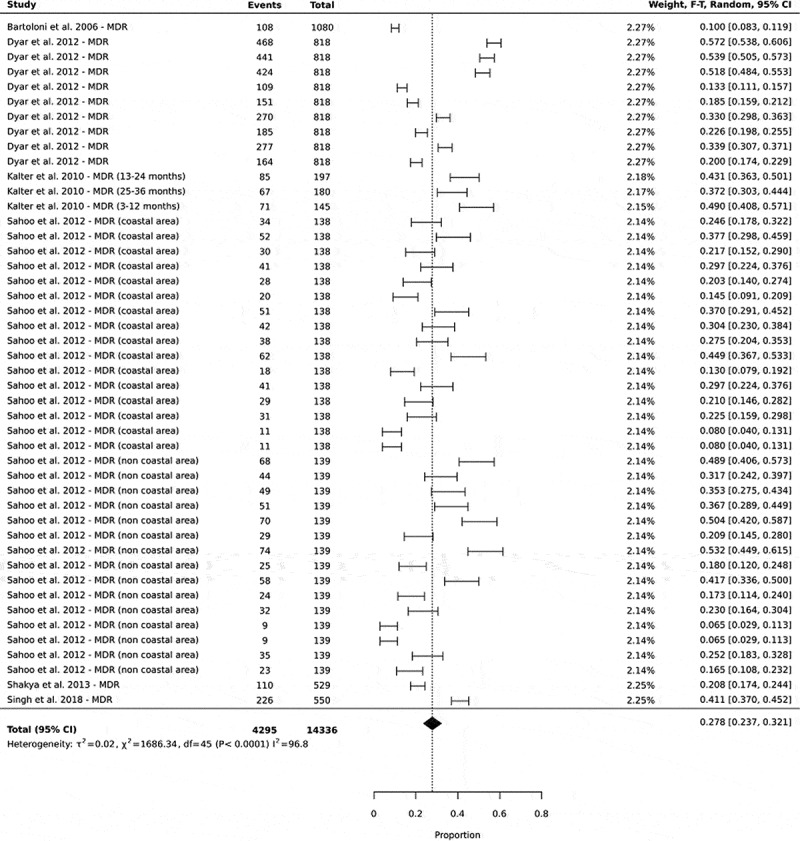
Forest plots showing the prevalence of multidrug-resistance in commensal *E. coli* isolated from human sources in community settings in low- and middle-income countries. In this analysis, the prevalence of the different antibiotic combinations in a single study were all included

In this study, multidrug-resistance was defined as the resistance of one or more agents in two out of the four different classes of antibiotics (protein, nucleic acid, cell wall, and folate synthesis). The pooled multidrug-resistance was 27% of 14,336 total cases of isolates tested, with 95% CI: 23–32 (Figure 2).

#### High prevalence of extensive-drug resistance (XDR) in community settings in low- and middle-income countries

**Figure 3.**
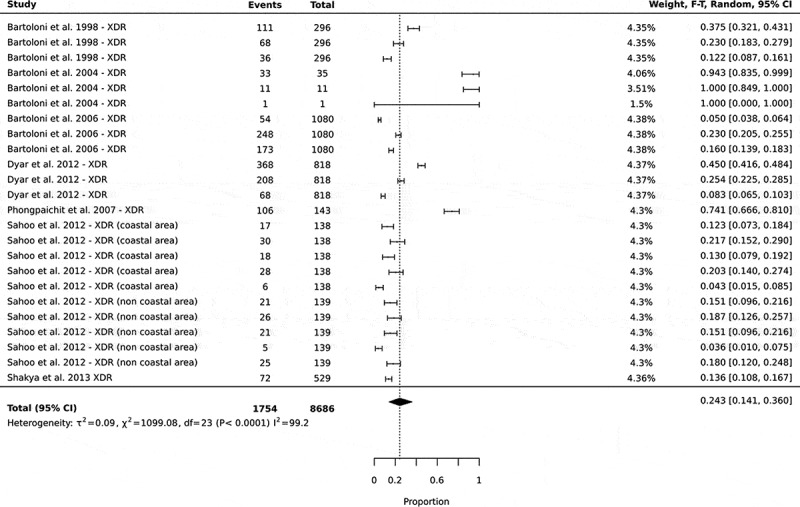
Forest plots showing the prevalence of extensive drug-resistance in commensal *E. coli* isolated from human sources in community settings in low- and middle-income countries. In this analysis, the prevalence of the different antibiotic combinations in a single study were all included

In this study, extensive-drug resistance was classified as the resistance of one or more agents in three out of the four different classes of antibiotics (protein, nucleic acid, cell wall, and folate synthesis). The pooled prevalence of extensive drug-resistance was 24% of 8686 total cases of isolates tested, with 95% CI: 14–36 (Figure 3).

### Emergence of pandrug-resistance (PDR) in community settings in low- and middle-income countries

**Figure 4.**
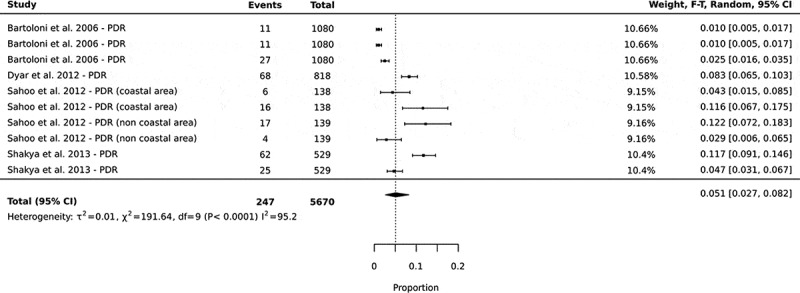
Forest plots showing the prevalence of pandrug-resistance in commensal *E. coli* isolated from human sources in community settings in low- and middle-income countries. In this analysis, the prevalence of the different antibiotic combinations in a single study were all included

Pandrug-resistance was defined as the resistance of one or more agents in all four classes of antibiotics (protein, nucleic acid, cell wall, and folate synthesis). Pandrug-resistance had a pooled prevalence of 5% of 5670 total cases of isolates tested, with 95% CI: 3–8 (Figure 4).

## Discussion

To contain the spread of multidrug-resistant bacteria, we must improve our knowledge of how multidrug-resistance is changing over time in LMICs, including the prevalence of multidrug-resistant commensal *Escherichia coli* in LMIC community settings (i.e. locations outside of, a hospital inpatient, acute care setting or a hospital clinic setting).

While there is an increase in multidrug-resistant pathogenic bacteria in general in community settings [41], we have observed that studies on multidrug-resistance of commensal *E. coli* isolated from healthy humans have consistently demonstrated very high incidences and prevalences in community settings in LMICs. This review synthesized eighteen studies that reported the prevalence of multidrug-resistance in commensal *E. coli* isolates from human sources in community settings in LMICs. Our results showed that the prevalence of multidrug- and extensive drug-resistance of *E. coli* at the community level is high in LMICs, and more concerning is the emergence of pandrug resistance in these settings. This evidence should prove useful to inform researchers, community pharmacists, and public health policymakers, among others, to develop appropriate interventions and to formulate and implement policies aimed at counteracting the global threats of multidrug-resistant infections and bacteria.

Most of the included studies had different definitions of multidrug-, extensive drug-, and pandrug-resistance, as also reported by Magiorakos et al. [42]. For this study, we classified resistance based on the inhibition mechanisms of action (protein, nucleic acid, cell wall, and folate synthesis inhibition). Multidrug-resistance was defined as the nonsusceptibility of at least one agent in two categories of the different mechanisms of action. At least one agent in three groups of the mechanism of action was classified as extensive drug resistance, and at least one agent in all four categories of the mechanism of action was classified as pandrug-resistance. As expected, the heterogeneity between studies was very high which may have been because several factors affect the prevalence of antibiotic resistance, such as previous antibiotic use [11,24,27,43], geographical location [26,32], age [24,26], socioeconomic status [30,32,43], and exposure to animals [27,44]. These factors were different in the included studies (Tables 1 and 2).

There are several drivers of antibiotic resistance whose inter-relationship is very complicated as it cuts across different sectors like health, agriculture, environment, and industry. The primary driver of multidrug-resistance in commensal *E. coli* in LMICs is the relaxed policies surrounding antibiotic access, prescription, and use [45]. Commensal *E. coli* are Gram-negative bacteria usually present in the guts of humans, animals, birds, and the environment [46]. When we ingest antibiotics for the treatment of bacterial infections, the commensal *E. coli* are exposed to these antibacterial agents and can develop resistance through natural selection [13]. Other drivers of multidrug-resistance in commensal *E. coli* in LMICs have been identified as inappropriate socioecological behaviors, poverty, overcrowding, lack of surveillance systems, food and supply chain safety issues, and highly contaminated waste effluents [45].

This study is in line with WHO recommendations, which call for improved awareness of the multidrug-resistance problem's existence as one of the five strategic objectives to control the problem spelled out in the Global action plan on antimicrobial resistance[47]. Many people in LMICs are aware of the existence of antibiotics, but fewer people are aware of antibiotic resistance [48]. Our findings will raise awareness of the extent of the problem and calls for urgent action to address this global health crisis especially in the current situation of the emergence of pandemics, like the severe acute respiratory coronavirus disease 2019 (COVID 19) that has led to a surge in the use of antimicrobial drugs within the community through self-medication and chemoprophylaxis. For COVID19, there has been an extensive use of not only antibiotics worldwide, but several medicines have also been tested in clinical trials as a potential treatment for COVID19 [49–57]. Such is the case for remdesivir, which has been authorised for the treatment of COVID19 patients even before approval [52]. A combination of both antibiotic and nonantibiotic drugs leads to an alteration of the gut microbiome that could fuel antibiotic resistance by commensal *Escherichia coli*, which can then be transmitted to other pathogenic enteric bacteria, e.g. *Salmonella typhi, Vibrio cholerae, Helicobacter pylori*, among others, through the exchange of mobile genetic materials [12,58–64].

Antibiotic resistance (ABR) is a growing problem that requires urgent action to stop its spread. Multiple interventions targeting different drivers of multidrug-resistance have been recommended [47]. Some of these interventions targets community settings and some are tailored to contribute to combating the challenges of multidrug-resistance. Based on the WHO antimicrobial control plan strategic objectives [47], the interventions are classified into the following categories; i) those aimed at improving awareness and understanding of antibiotic resistance, for example, it is recommended that creating participatory videos (participatory documentaries) about antibiotic resistance would be useful in creating awareness in communities [65]; ii) activities strengthening knowledge through active and routine surveillance and research; iii) those focussing on the reduction of infections for example improvement in sanitation and hygiene, to reduce the demand of antibiotics; iv) those that optimize the use of antibiotics, for example, the formulation and implementation of regulations surrounding the use of antibiotics coupled with strict monitoring of antibiotic use as part of the policy, in addition to the development of new screening and diagnostic tools [66]; and v) interventions ensuring that there is a sustainable investment in combating antibiotic resistance. A global and interdisciplinary approach will fast track implementation of some of the interventions that may be universal, for example, the development of new screening and diagnostic tools [66]. The recommended interventions have shown to have an impact on the combating of multidrug-resistance at the community level. However, in order to harness the community’s potential in combating this problem, active community engagement and involvement from the design to the implementation of the selected intervention should be emphasized and carried out in order to allow the implementation of interventions that are well adapted to the local context and easy to scale up at less cost [67].

To conclude, this study provides an appraisal of the evidence on the high prevalence of multidrug-, extensive drug resistance, and the emergence of pandrug-resistant commensal *E. coli* from healthy human sources in community settings in LMICs. This evidence appraisal calls for urgent action in the fight against multidrug-resistance in community settings to make antibiotic resistance history.

## Paper context

Studies conducted to investigate the prevalence of multi-drug resistance in commensal *Escherichia coli* isolated from humans in community settings in low- and middle-income countries have consistently been high. This study provides an appraisal of the evidence on the high prevalence of multidrug-resistance in commensal *E. coli* isolated from humans in community settings in low- and middle-income countries. Our findings call for greater effort to be placed at the community level to reverse the course of multidrug-resistance.
